# Interferons and *Toxoplasma gondii* shape PD-L1 regulation in retinal barrier cells: the critical role of proteases

**DOI:** 10.3389/fimmu.2025.1607247

**Published:** 2025-06-17

**Authors:** Benjamin Geiller, Camila Cevallos, Iuliia Tsybenko, Lydia Arnoux, Marie-Paule Felder-Schmittbuhl, Alexander W. Pfaff

**Affiliations:** ^1^ UR 3073 Pathogens Host Arthropods Vectors Interface, Toxoplasmosis Immunology Team, Université de Strasbourg, Strasbourg, France; ^2^ Centre National de la Recherche Scientifique, Institut des Neurosciences Cellulaires et Intégratives (UPR 3212), Université de Strasbourg, Strasbourg, France; ^3^ Service de Parasitologie et Mycologie Médicale, Hôpitaux Universitaires de Strasbourg, Strasbourg, France

**Keywords:** *Toxoplasma gondii*, ocular toxoplasmosis, interferons, PD-L1, retina, immune privilege

## Abstract

**Introduction:**

The apicomplexan parasite *Toxoplasma gondii* establishes chronic infection in the central nervous system, including the retina, causing ocular toxoplasmosis (OT). This persistence relies on a fine balance between inflammatory and immunomodulatory mechanisms, especially in the immune-privileged ocular environment. We previously described the immunologic interactions between retinal cells, and particularly the roles of type I and III interferons. In this study, we investigated the regulatory dynamics of PD-L1, a central immunomodulatory receptor on immune cells.

**Methods:**

We first investigated the mechanisms of PD-L1 regulation and the roles of type I and III interferons in an *in vitro T. gondii* infection model using mono- and co-culture systems of human microglia, astrocytes, and Müller cells. We also assessed PD-L1 expression in an outer blood-retina barrier model (oBRB) of differentiated retinal pigmented epithelial (RPE) cells. Additionally, we looked at retinal cell activation, PD-L1 expression and the roles of these interferons in a mouse model of OT.

**Results:**

Our findings reveal new roles for type I and III interferons in regulating glial cell activation and PD-L1 expression in RPE, Müller, astrocytes and microglial cells. Notably, Müller cells, the most abundant glial cells in the retina, showed the highest baseline PD-L1 expression at both the mRNA and protein levels, and responded robustly to interferon stimulation. This points to a more prominent immunoregulatory role for Müller cells in the retina than previously recognized. Furthermore, we identified a parasite protease-dependent mechanism that reduces PD-L1 expression in our *in vitro* oBRB model potentially contributing to immune evasion and inflammation during OT. Finally, in a murine model of OT, we demonstrated that PD-L1 expression reached its peak on day 7 post-infection and that interferon neutralization plays a crucial role in regulating both PD-L1 expression and glial activation.

**Discussion:**

The parasite *T. gondii* orchestrates the IFN type I and III dependent retinal immune interaction and downregulates PD-L1 in the oBRB by a protease-dependent mechanism, potentially contributing to immune evasion and inflammation in retinal infection. Our results can pave the way to fully elucidate retinal immune networks and PD-L1 regulation mechanisms, offering potential targets for therapeutic interventions in OT and other retinal inflammatory diseases.

## Introduction

The apicomplexan parasite *Toxoplasma gondii* establishes chronic infection in the central nervous system, including the retina, causing ocular toxoplasmosis (OT) (1). This persistence relies on a fine balance between inflammatory and immunomodulatory mechanisms, especially in the immune-privileged ocular environment, where immune reactions must be tightly regulated to prevent irreversible damage. A central immunomodulatory receptor on immune cells is PD-1 (Programmed cell death protein 1), which binds to its cognate ligand PD-L1 (*CD274*) (2). In a mouse model of OT, PD-L1 was shown to be expressed by multiple infiltrating and resident retinal cell types, where it effectively inhibited CD4+ T cell proliferation (3). In this model, PD-L1 upregulation was critically dependent on interferon gamma (IFN-γ), a central mediator of the immune response against *T. gondii* (4). In addition to IFN-γ, which is the sole representative of type II interferons, two other classes of interferon exist: type I interferons, primarily represented by IFN-α and IFN-β, and type III interferons, which include the IFN-λ isotypes (5). The expression of PD-L1 induced by interferons predominantly depends on the transcription factor interferon regulatory factor 1 (IRF1), an interferon-stimulated gene (ISG) that is specifically upregulated by type I and II interferons (6, 7). However, recent studies have shown that PD-L1 expression is enhanced in non-small cell lung cancer cells in response to type III interferons (8), raising questions about the roles of type I and III interferons in regulating PD-L1 expression in retinal cells during OT.

Although the roles of type I and III interferons in *T. gondii* infection have not been fully described, we previously demonstrated that both types enhance the barrier function of the outer blood-retinal barrier (oBRB) by regulating the tight junction localization of the chaperone protein zonula occludens-1 (ZO-1) (9). However, the impact of the ocular environment during infection on both barrier function and immune regulation remains unclear. This is particularly important, as uncontrolled inflammation can impair oBRB function (10).

In this study, we investigated the mechanisms of PD-L1 regulation in an *in vitro T. gondii* infection model using mono- and co-culture systems of human microglia, astrocytes, and Müller cells. We also assessed the effects of PD-L1 expression in an oBRB model composed of differentiated retinal pigmented epithelial (RPE) cells. Additionally, we used a mouse model of OT to assess the roles of type I and type III interferons in glial activation, PD-L1 expression, and microglial activity.

Our results revealed new roles for type I and III interferons in regulating PD-L1 in different retinal cell types during *T. gondii* infection. Furthermore, we identified a parasite protease-dependent mechanism that reduces PD-L1 expression, potentially disrupting immune regulation and promoting a strong local inflammatory response within the retina.

## Materials and methods

### Cell lines and parasites

The HMC3, U-118MG and ARPE-19 cell lines present in this study were obtained from the American Type Culture Collection (ATCC). The human Müller cell line Moorfields/Institute of Ophthalmology- Müller 1 (MIO-M1) was obtained from the UCL Institute of Ophthalmology, London, UK (11). Astrocytes U-118MG, Müller MOI-M1 and Microglia HMC3 were cultivated in DMEM (Gibco), supplemented with 4.5g/L L-glutamine (Gibco), 10% inactivated FBS (Gibco) and P/S (Hyclone). Human RPE ARPE-19 cells were differentiated according to the protocol described by Hazim et al. (12). Briefly, cells were cultivated in T75 flask (Falcon) for two weeks with MEM-Nicotinamide medium: 1% N1 supplement (Invitrogen), 1% inactivated FBS (Gibco), P/S (Hyclone), 0.25mg/mL Taurine (Sigma-Aldrich), Hydrocortisone 20ng/mL (Sigma-Aldrich), Triiodo-thyronine 0.013 ng/mL (Sigma-Aldrich) and 10mM nicotinamide (Sigma Aldrich). After 3 times 10min TrypLE (Gibco) treatment, the remaining cells were placed on 0.4µM PET transwell inserts (Falcon, 353095) in 24-well-plates (Falcon), coated with natural mouse laminin (Invitrogen). Cell medium was changed 3 times per week during 8 weeks before use.

All cell incubations were performed at 37°C and 5% CO_2_. The *T. gondii* strains RH and Me49 were obtained from the French Biological Resource Center *Toxoplasma* (CRB *Toxoplasma*; Laboratoire de Parasitologie, CHU Reims, Reims, France). Parasites were maintained by passages in Vero cells (ATCC) in RPMI 1640 (Gibco) without L-glutamine, 10% inactivated FBS (Gibco), P/S (Hyclone). Before use, parasites were washed three times in cold PBS, filtered through a 5µm filter (Millex: SLSV025LS) and counted by trypan blue exclusion on a KOVA slide.

### RT-qPCR

5 x 10^4^ astrocytes, microglia, or Müller cells were cultivated for 48 hours until near confluency in 24 well plates (Nunc). For RPE cells, 8-week-differentiated cells were used. Dependent on experimental conditions, cells were treated with 20ng/mL human recombinant IFN-β, IFN-γ or IFN-λ1 (Peprotech) or infected with a multiplicity of infection (MOI) of 1 parasite for 1 cell (1:1) corresponding to approximately 3 x 10^5^ RH or Me49 *T. gondii* tachyzoites for all cell types. After 24 hours, cells were harvested with Nucleozol (Macherey-Nagel) reagent and total RNA extracted following manufacturer’s recommendation. RNA quantification and quality check were performed by measuring the OD at 260/280/230 nm by µdrop with a Varioskan LUX (Thermo Scientific). 10ng of RNA were reverse transcribed to cDNA, using qScript™ cDNA Synthesis Kit (Quantabio, 733-1174) following manufacturer’s recommendation. Then, Real-time PCR specific for PD-L1 (Forward: *5’-TTGCTGAACGCCCCATACAA-3’*; Reverse: *5’-GTAGCCCTCAGCCTGACATC-3’*) and housekeeping gene GAPDH (Forward: *5’-AGCAATGCCTCCTGACCACCAAC -3’*; Reverse: *5’-CCGGAGGGGCCATCCACAGTCT -3’*) mRNA was performed on CFX light cycler and SSoAdvanced SybrGreen (Biorad). Finally, fold changes were calculated using the 2-ΔΔCT method.

### Western blot

8 x 10^5^ astrocytes, microglia or Müller cells were plated in 10mL petri dishes (TPP) for 48h. For RPE cells, 8 week differentiated cells were used. Cells were infected with a MOI of 1:1 corresponding to approximately 3 x 10^6^ RH or Me49 *T. gondii* tachyzoites for 24 hours. Subsequently, cells were washed in 1X TBS (Thermo Scientific) and harvested on ice using a cell scrapper (Falcon) and RIPA buffer composed of: 25mM Tris HCl pH 7,6; 150mM NaCl; 1% NP-40; 1% sodium désoxycholate; 0.1% SDS; 0.01% Triton x-100; 1 protease/phosphatase inhibitor tab for 10mL (Pierce). Cell lysis and protein extraction were then completed by 1h incubation on ice, followed by 2 rapid freeze/thaw cycles at -80°C.

To perform the biphasic extraction of cytosolic and membrane proteins, the Mem-PER Plus Kit (Thermo Scientific 89842Y) was used following manufacturer’s recommendations.

Lysates were then denatured at 100°C for 10 minutes in 2X Laemmli buffer (Biorad) containing 10% β-mercapto-ethanol and proteins separated in a 10% SDS-PAGE at 150V for 1h using an Novex mini gel tank and blot module. Proteins were then transferred on a polyvinylidene difluoride PVDF membrane (Biorad) at 30V for 1h. Wash steps were made using 1X TBS containing 0.05% Tween-20. Membranes were then blocked for 2h with 1X TBS and 5% BSA at room temperature (RT), They were then incubated with mouse@PD-L1 (Invitrogen, 14-5983-82) at 500ng/mL or mouse@β-actin at 50ng/mL (Invitrogen, A5441) antibodies overnight at 4°C. Finally, membranes were incubated 2h at RT with 125ng/mL sheep anti-mouse IgG HRP (GE healthcare, NA931V) secondary antibodies and visualized with clarity western ECL substrate (Biorad) on ChemiDoc MP (Biorad). Band intensities were analyzed using FIJI gel tools.

### Immunofluorescence staining of glial and microglia cells

5 x 10^4^ astrocytes, microglia, or Müller cells were cultivated on round borosilicate glass cover slips for 48 hours until near confluency in 24 well plates (Nunc). Dependent on experimental conditions, cells were treated with 20ng/mL human recombinant IFN-β, IFN-γ or IFN-λ1 (Peprotech) or infected with a MOI of 1:1 corresponding to 3 x 10^5^ RH or Me49 *T. gondii* tachyzoites. After 24 hours, cells were first fixed with 4% paraformaldehyde (PFA) in PBS for 15 minutes and then permeabilized with PBS + 0.1% Triton X-100 for 10 minutes at RT. Then, cells were incubated for 2h in PBS + 5% BSA at RT, followed by incubation with 5µg/mL Rat@PD-L1 ab (Invitrogen 14-5982-82) overnight at 4°C. Finally, cells were incubated during 1h at RT with 2.5 µg/mL donkey@rat ab linked to AlexaFluor647 (Invitrogen A48272) and 2 min with 1/200 diluted Hoechst33258 (Sigma 94403), before being mounted on microscopy slides with Prolong Gold anti-fade reagent (Invitrogen). Images were taken with a Zeiss Apotome 3 epifluorescence microscope. Settings were defined at the beginning of each experiment with the first control sample and maintained for all other samples of the same replicate. Image post treatment and measurements were made with FIJI software.

### Coculture supernatant

Astrocytes, microglia and Müller cells were cocultured during 48 hours in DMEM (Gibco), 4.5g/L L-glutamine (Gibco), 10% inactivated FBS (Gibco), P/S (Hyclone) at a 1:1:1 ratio with a total amount of 5 x 10^4^ cells per well in 24 well plates (Nunc). Homogeneous plating and correct cell ratio were verified with an inverted brightfield microscope with phase contrast. Cocultures were then infected at a MOI of 1:1 corresponding to approximately 3 x 10^5^ RH or Me49 *T. gondii* tachyzoites for 20 hours. Control cultures were left non-infected. Finally, coculture media were harvested, centrifuged at 5000 x g during 15min at 4°C and supernatants stored at -80°C until use.

### Parasite supernatant

10^7^ *T. gondii* tachyzoites per well were placed in a 24 well plate (Nunc) in 200µL DMEM (Gibco), 4.5g/L L-glutamine (Gibco), 10% inactivated FBS (Gibco), P/S (Hyclone) for 24 hours. Finally, the culture media was harvested, centrifuged at 5000 x g during 15min at 4°C and supernatants stored at -80°C until use. For some experiments, parasite supernatants were treated with a protease inhibitor cocktail suitable for cell culture (Sigma, P1860-1ML) at 1/200 dilution just before use, as recommended by the manufacturer for effective protease inactivation without toxicity.

### Effect of coculture supernatant and parasite supernatant on oBRB model

Differentiated RPE cells were treated with coculture supernatants or parasite supernatants, depending on experimental conditions. Conditioned medium was placed in both the upper transwell compartment (100 µL) and the bottom well (600 µL). For infected conditions, RPE cells were infected with a MOI of 1:1 corresponding to 3 x 10^5^ RH or Me49 *T. gondii* tachyzoites. After 20 hours of incubation, cells were washed three times with 1X PBS, fixed with 4% PFA for 15 minutes and then permeabilized with PBS + 0.1% Triton X-100 for 10 minutes at RT. Then, cells were incubated for 2h in PBS + 5% BSA at RT followed by an overnight incubation at 4°C with 5µg/mL rat@PD-L1 (Invitrogen 14-5982-82) and 5µg/mL mouse@ZO-1 (invitrogen 33-9100). Cells were then incubated for 1h at RT with 2.5 µg/mL donkey@rat AlexaFluor647 ab (Invitrogen A48272) and 2.5 µg/mL sheep@mouse AlexaFluor555+ ab (Invitrogen A32727), and finally 2 minutes with 1/200 diluted Hoechst33258 (Sigma 94403). Then, the membranes were cut out using a scalpel before being mounted on microscopy slide with Prolong Gold anti-fade reagent (Invitrogen). Images were taken with a Zeiss LSM 800 Airyscan confocal microscopy system. Settings were defined at the beginning of each experiment with the first control sample and maintained for all other samples of the same replicate. Image post treatment and measurements were made with FIJI software. The measurement of specific ZO-1 tight junction fluorescence was realized as follows (https://imagej.net/imaging/segmentation): First, auto threshold (method=default white) was applied, then the image was converted to mask and copied by the tool “Create selection”. This selection was then restored on the raw image and the mean gray values and area data were gathered. The same protocol was applied to all images.

### Mouse infection

Animal experimentation was approved by the local ethics committee (French national authorization number: APAFIS#43895-2023061311453553). Female SWISS mice were purchased by Janvier Labs (Le Genest St Isle, France) and housed in our SPF facility (accreditation number E-67-482-34) until use. Eight-to-twelve-week-old mice were intravitreally injected in both eyes using a 30-gauge needles (BD Microlance) on a 10µL syringe (Hamilton) under isoflurane anesthesia. Depending on experimental condition, the injected 5µL of sterile PBS per eye contained 20ng of mouse IgG1 kappa isotype control P3.6.2.8.1 (Invitrogen 14-4714-82) or mouse IL-28A/B neutralizing antibody (Biotechne, MAB17892-100) or mouse IFN-β neutralizing antibody (Biotechne, MAB8234-100). In infected conditions, 4,000 *T. gondii* Me49 tachyzoites were added to the solution. Three mice per group were euthanized on days 1, 3 and 7 post injection for immunofluorescence applications.

### Mouse retina immunofluorescence

Eyes were enucleated and immediately fixed overnight at 4°C in 4% PFA in PBS. Eyes were then washed in PBS and incubated in sucrose gradient in PBS as follows: 2h in 10% sucrose, overnight at 4°C in 20% sucrose, 2h in 30% sucrose. Then, they were embedded in Tissue-Tek OCT compound (Sakura Fintek), snap frozen in liquid nitrogen and stored at -80°C. Using a cryostat (Leica), 16 µm thick sections were cut, placed on SuperfrostPlus adhesion microscope slides (Epredia, J1800AMNZ) and stored at -20°C.

For immunostaining, cryosections were incubated for 2h in PBS + 5% BSA at RT followed by overnight incubation at 4°C with 1.25 µg/mL rat@PD-L1 (Invitrogen 14-5982-82), 1.25 µg/mL mouse@CD11b (Invitrogen) and 1.25 µg/mL rabbit@GFAP (Invitrogen). Then, sections were incubated for 1h at RT with 1 µg/mL donkey@Rat AlexaFluor647 (Invitrogen A48272), 1 µg/mL sheep@mouse AlexaFluor555+ (Invitrogen A32727) and 1 µg/mL sheep@rabbit AlexaFluor444 (Invitrogen) and 2 minutes with 1/200 diluted Hoechst33258 (Sigma 94403). Finally, coverslips were mounted with Prolong Gold anti-fade reagent (Invitrogen). Images were taken with a Zeiss LSM 800 Airyscan confocal microscopy system. Settings were defined at the beginning of each experiment with the first control sample and maintained for all other samples of the same replicate. Image post treatment and measurements were made with FIJI software.

### Statistical analysis

Statistical analyses were performed using GraphPad Prism version 8.0. First, normality and equal standard deviation were analyzed by a Shapiro-Wilk test. When sampling followed normal distribution, the appropriate parametric test was performed. Otherwise, the corresponding non-parametric test was performed. If the result is normalized to a control group and only two groups were compared, a parametric one sample t test or corresponding non-parametric Wilcoxon signed-ranked was performed with a theorical mean of 1. If the values were absolute values and more than two groups were compared, a One-Way ANOVA test with multiple comparison correction was performed. Finally, if only two groups with absolute values were compared, a parametric Student’s *t-*test or corresponding non-parametric Mann-Withney test was performed. For all statistical analyses, *p*-values < 0.05 were considered significant.

## Results

### Infection increases PD-L1 membrane protein levels in microglial cells, with opposite effects on RPE cells

**Figure 1:**
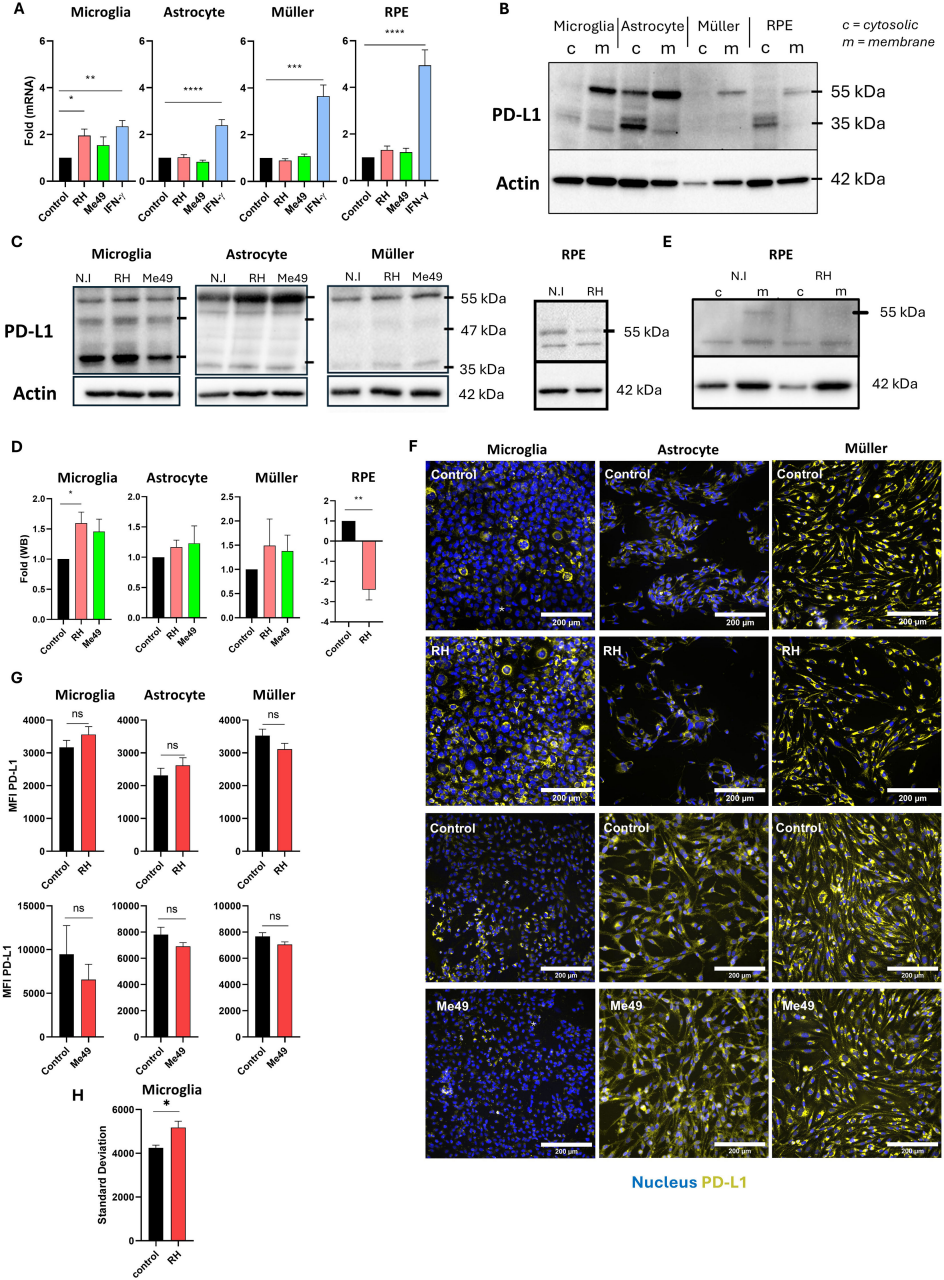
Modulation of PD-L1 mRNA and protein expression by *T. gondii* infection **(A)** Quantitative RT-qPCR analysis of PD-L1 mRNA expression levels in human microglia, astrocytes, Müller cells, and RPE cells following infection with *Toxoplasma gondii* RH or Me49 strains, or with 20 ng/mL IFN-γ. Data are presented as mean ± SEM of fold changes, normalized to the GAPDH housekeeping gene and to non-infected/non-stimulated controls. Results are pooled from three independent experiments, each with three replicates (n=9). Statistical analysis was performed using one-way ANOVA; *P < 0.05, **P < 0.01, ***P < 0.001, ****P < 0.0001 compared to control. **(B)** Western blot analysis showing PD-L1 and β-actin expression in membrane (m) and cytosolic **(c)** protein fractions isolated from human microglia, astrocytes, Müller cells, and RPE cells. **(C)** Western blot analysis of PD-L1 and β-actin expression in whole-cell lysates from human microglia, astrocytes, Müller cells, and RPE cells infected with *T. gondii* RH or Me49 strains (MOI 1:1). **(D)** Quantification of PD-L1 band intensity from western blots, normalized to β-actin and non-infected controls. Data are shown as mean ± SEM from three independent experiments (n=4). Statistical analysis: one-way ANOVA; *P < 0.05, **P < 0.01 compared to control. **(E)** Western blot analysis of PD-L1 and β-actin in membrane (m) and cytosolic (c) fractions from 8-week differentiated RPE cells infected with *T. gondii* RH. The experiment was repeated three times with consistent results. **(F)** Representative fluorescence microscopy images of PD-L1 expression in human astrocytes, microglia, and Müller cells infected with virulent *T. gondii* RH or less-virulent *T. gondii* Me49 strain (MOI 1:1). PD-L1 is shown in yellow, and nuclei are stained with Hoechst 33342 in blue. Images represent one of three independent replicates showing similar results. **(G)** Mean fluorescence intensity (MFI) analysis of PD-L1 expression in human microglia, astrocytes, and Müller cells infected with virulent *T. gondii* RH or less-virulent Me49 strain (MOI 1:1). Data are presented as mean ± SEM from three independent experiments (n=12). Statistical analysis was performed using a T-test; ns = not significant (P > 0.05), *P < 0.05 compared to control. **(H)** Analysis of PD-L1 fluorescence variability (standard deviation) reflecting average variability within each image. Data are presented as mean ± SEM from three independent experiments (n=12). Statistical analysis: one-way ANOVA; *P < 0.05 compared to control.

Although PD-L1 expression has been reported in the context of OT in mice, its expression by human retinal cell types during *T. gondii* infection has not been explored. To address this, we infected human microglia, astrocytes, Müller cells and RPE cells with either the virulent type I *T. gondii* RH strain or the less virulent type II Me49 strain for 24h, and measured PD-L1 mRNA levels by real-time RT-qPCR. Relative expression levels were compared to a non-infected control, with IFN-γ stimulation serving as a positive control due to its well-documented ability to induce PD-L1 expression in various cell types. Our findings reveal that infection with either the RH or Me49 strain did not significantly alter PD-L1 mRNA expression in astrocytes, Müller cells, or RPE cells (**Figure 1A**). By contrast, infection with the RH strain significantly increased PD-L1 mRNA expression in microglial cells, reaching levels comparable to those induced by IFN-γ stimulation. Although Me49 infection also showed a trend toward increased PD-L1 expression in microglia, this effect was not statistically significant. Given the potential post-translational modifications of PD-L1 that affect protein stability and cellular localization, we next examined PD-L1 protein levels and membrane localization. PD-L1 is a highly glycosylated membrane protein, resulting in multiple bands on Western blot ranging from 33 kDa to approximately 55 kDa. Higher molecular weight forms correspond to more glycosylated membrane-associated proteins, while lower molecular weight bands reflect non- or low-glycosylated cytosolic forms (13). To distinguish these forms, we performed differential extraction of membrane and cytosolic proteins. In all cell lines, the 55 kDa form was enriched in the membrane fraction, particularly in RPE, microglia, and Müller cells, where no 55 kDa PD-L1 protein was detected in the cytosolic fraction. By contrast, the 35 kDa form predominated in the cytosolic fraction (**Figure 1B**). Therefore, in subsequent experiments, we considered the 55 kDa form as the active, membrane-localized form of PD-L1.

We then investigated PD-L1 protein expression following infection with *T. gondii* RH and Me49 strains. In microglial cells, infection with the RH strain slightly (*p*=0.047) increased the intensity of the 55 kDa band. Consistent with the mRNA results, infection with Me49 also slightly increased PD-L1 protein levels, though this change was not statistically significant (**Figures 1C, D**). Infection did not significantly impact PD-L1 protein expression in astrocytes or Müller cells. Interestingly, infection significantly reduced PD-L1 expression in RPE cells (**Figures 1C, D**), particularly affecting the higher 55 kDa molecular weight band. Indeed, upon differential extraction of membrane and cytosolic proteins, the 55 kDa PD-L1 form was absent in the membrane fraction following infection (**Figure 1E**).

Immunofluorescence microscopy did not show apparent changes in membrane PD-L1 expression and mean fluorescence intensity in astrocytes and Müller cells (**Figures 1F, G**). Interestingly, for microglial cells, fluorescence images showed the appearance of PD-L1^low^ cells following RH infection, while mostly PD-L1^high^ cells were visible in uninfected cells (**Figure 1F**). This did not alter MFI values (**Figure 1G**), but led to a significantly increased heterogeneity in MFI values of infected cells (**Figure 1H** **).** This response appeared to be specific to microglia and may be attributed to the presence of a negative paracrine feedback loop regulating PD-L1 expression.

### Type I and II, but not type III, interferons enhance PD-L1 mRNA expression in human glial and RPE cells

**Figure 2:**
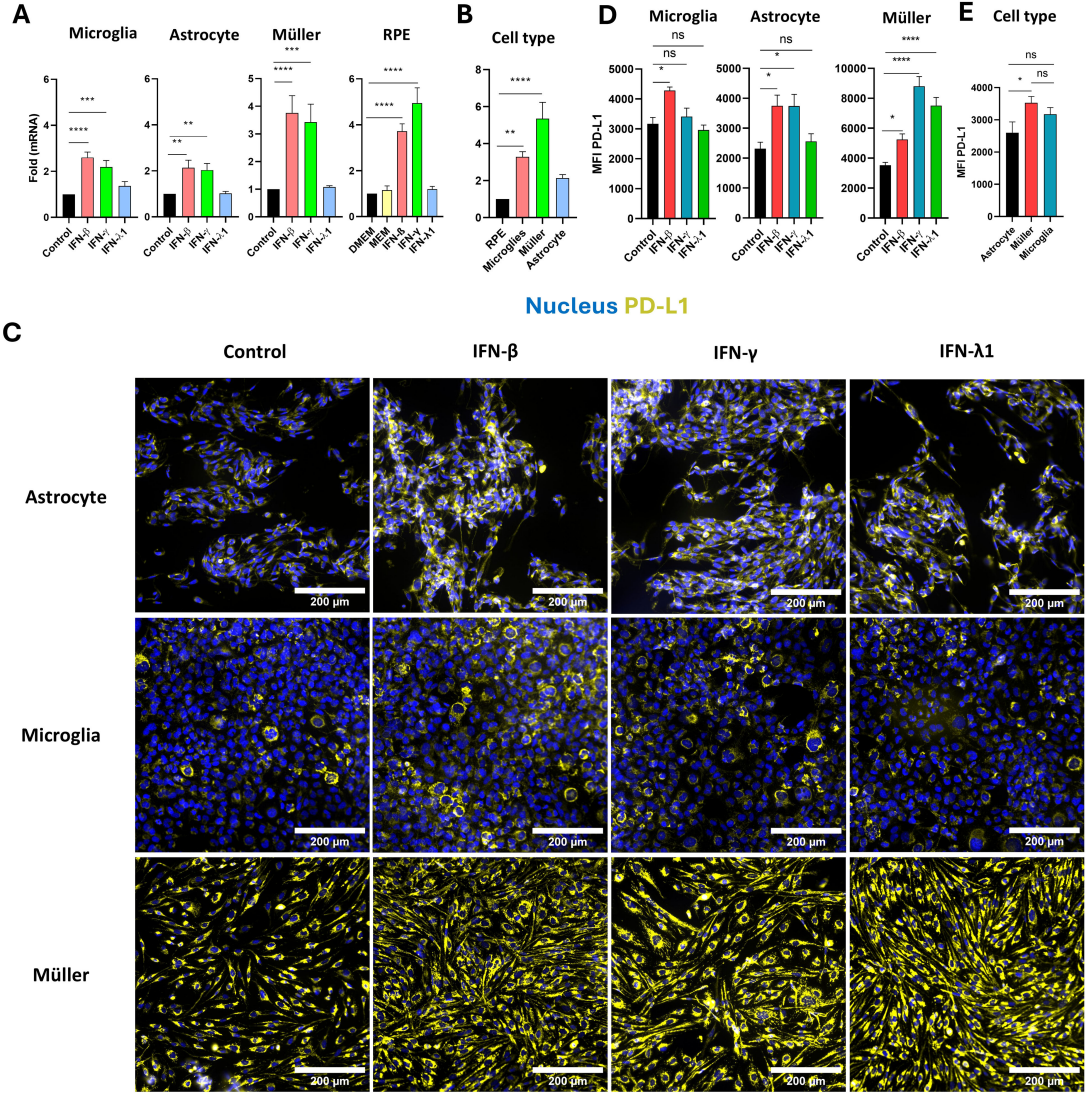
Regulation of PD-L1 in human microglia, astrocytes, Müller, and RPE cells by IFN stimulation. **(A)** RT-qPCR analysis of PD-L1 mRNA expression in human microglia, astrocytes, Müller cells, and RPE cells following stimulation with 20 ng/mL IFN-β, IFN-γ, or IFN-λ1. Data are presented as mean ± SEM from four independent experiments, each with four replicates (n=12). Statistical analysis was performed using one-way ANOVA; *P < 0.05, **P < 0.01, ***P < 0.001, ****P < 0.0001 compared to control. **(B)** Baseline PD-L1 mRNA expression in human microglia, astrocytes, Müller cells, and RPE cells measured by RT-qPCR. Results are shown as mean ± SEM of fold changes normalized to GAPDH and to RPE cells, which exhibited the lowest PD-L1 expression. Statistical analysis: one-way ANOVA; *P < 0.05, **P < 0.01, ***P < 0.001, ****P < 0.0001 compared to RPE cells. **(C)** Representative fluorescence microscopy images of PD-L1 expression in human astrocytes, microglia, and Müller cells following stimulation with 20 ng/mL IFN-β, IFN-γ, or IFN-λ1. PD-L1 is shown in yellow, nuclei in blue using Hoechst 33342. Images represent one of three independent replicates demonstrating consistent results. **(D)** Mean fluorescence intensity (MFI) analysis of PD-L1 expression in human microglia, astrocytes, and Müller cells following stimulation with 20 ng/mL IFN-β, IFN-γ, or IFN-λ1. Data are shown as mean ± SEM from three independent experiments (n=6 for IFN stimulation; n=12 for infection). Statistical analysis: one-way ANOVA; *P < 0.05, **P < 0.01, ***P < 0.001, ****P < 0.0001 compared to control. **(E)** Baseline analysis of PD-L1 MFI in human microglia, astrocytes, and Müller cells. Data are expressed as mean ± SEM from three independent experiments (n=6). Statistical analysis: one-way ANOVA; ns = not significant (P > 0.05), *P < 0.05 compared to control.

During the acute phase of *T. gondii* infection, high levels of interferons are secreted, playing a critical role in the immune response. We examined the effects of IFN-β, IFN-γ, and IFN-λ1 on PD-L1 mRNA expression in human retinal cells by real-time RT-qPCR analysis. Consistent with prior findings, IFN-γ robustly induced PD-L1 mRNA expression across all studied cell types (**Figure 2A**). Type I IFN-β also significantly increased PD-L1 expression to a level comparable to IFN-γ. By contrast, type III IFN-λ1 had no observable effect on PD-L1 mRNA expression in any cell type. Interestingly, Müller and RPE cells were the most responsive to interferon stimulation compared to microglia and astrocytes. On average, interferons induced a twofold increase in PD-L1 expression in microglia and astrocytes, while Müller and RPE cells exhibited an approximately fourfold increase. Despite this strong response, RPE cells exhibited the lowest baseline PD-L1 expression (cell-specific PD-L1 expression without any stimulation) compared to the other cell types. Specifically, compared to RPE cells, baseline PD-L1 expression was approximately 4-fold higher in microglia, 6-fold higher in Müller cells, and 2-fold higher in astrocytes (**Figure 2B**). This baseline expression is particularly important for an immune-regulating ligand such as PD-L1, as it reflects its steady-state capacity to inhibit lymphoid activation.

Interferon-induced mRNA expression also influenced PD-L1 protein level and membrane localization. Fluorescence imaging showed a significant increase in PD-L1 expression in astrocytes, microglia and Müller cells in response to IFN-β, in Müller cells and astrocytes in response to IFN-γ, and exclusively in Müller cells in response to IFN-λ1 (**Figures 2C, D**). For microglial cells, treatment with IFN-γ, which is pro-inflammatory, also not significatively increase MFI but elevate the number of PD-L1^high^ microglial cells, confirming previous observations from RH infection. Interestingly, in astrocytes, and even more prominently in Müller cells, all types of interferons increased PD-L1 membrane localization and distribution. PD-L1 labeling was detected across the entire cell surface and cytoplasmic extensions in stimulated cells, whereas in non-stimulated cells, it was localized around the cell body near the nucleus (**Figure 2C**). Notably, Müller cells were the only cell type responsive to IFN-λ1 stimulation at the protein level, despite the absence of mRNA induction (**Figure 2A**), suggesting that IFN-λ1 induces PD-L1 membrane relocalization in Müller cells, but not in microglia and astrocytes. Consistent with their higher PD-L1 mRNA expression, Müller cells exhibited significantly stronger baseline PD-L1 protein labeling compared to astrocytes, which expressed the lowest levels of PD-L1 (**Figure 2E**). Furthermore, Müller cells were the most responsive to interferon treatment, especially IFN-γ, with an average MFI of 8807 arbitrary units (AU), in contrast to astrocytes and microglia, which had average MFIs of 3738 AU and 3410 AU, respectively.

These findings demonstrate that type I and II interferons effectively induce PD-L1 mRNA expression in RPE cells, astrocytes, Müller cells, and microglia. Particularly, Müller cells exhibit the highest PD-L1 expression and responsiveness to interferons and are the only cells showing enhanced MFI by redistribution of PD-L1 to the membrane in response to IFN-λ1. These results suggest a more substantial role for Müller cells in PD-L1-mediated immune regulation within the retina than previously recognized.

### Excreted parasitic proteases drive PD-L1 degradation in RPE cells of the outer blood-retinal barrier

**Figure 3:**
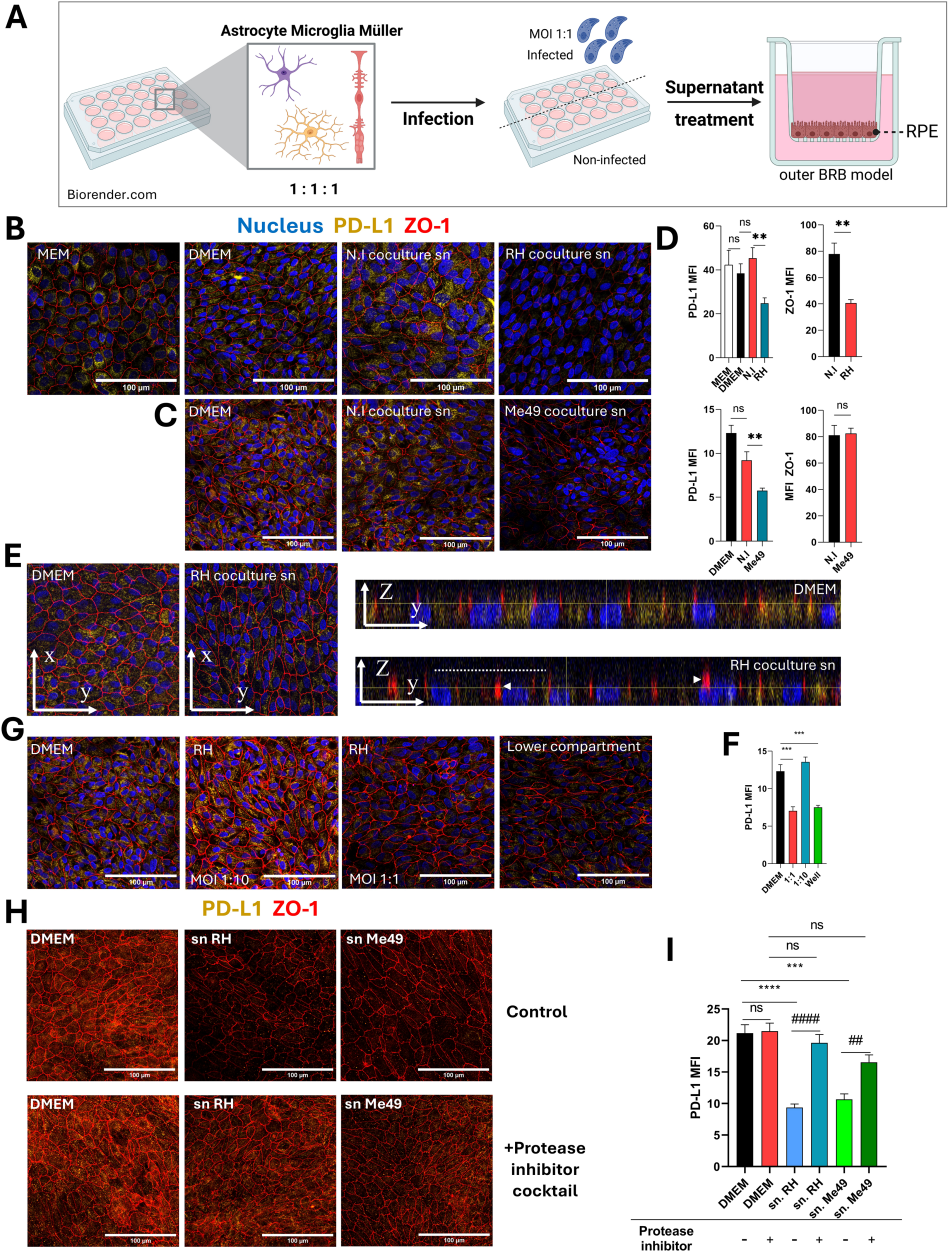
Protease-dependent reduction of PD-L1 expression on the outer blood-retinal barrier model by infected co-culture supernatants. **(A)** Schematic diagram of the experimental protocol. Astrocytes, microglia, and Müller cells are co-cultured for 48 hours, followed by infection with *T. gondii* RH or Me49 tachyzoites for 20 hours. Culture media is then centrifuged to remove parasites, and the supernatants are collected. The oBRB (outer blood-retinal barrier) model is treated with these supernatants for 24 hours, after which PD-L1 and ZO-1 expression are analyzed via confocal microscopy. Created in BioRender. Geiller, **(B)** (2025) https://BioRender.com/n37e493. **(B, C)** Confocal images of the oBRB model illustrating the effects of infected co-culture supernatants on ZO-1 tight junction localization (red) and PD-L1 expression (yellow). Cell nuclei are stained in blue. MEM-Nic is the medium used for RPE cell differentiation, while DMEM is the medium used to produce co-culture supernatants (sn). N.I refers to non-infected co-cultures, and *T. gondii* RH or Me49 denotes infected co-cultures. Images represent one of three independent replicates with similar results. **(D)** Mean fluorescence intensity (MFI) analysis of confocal images, quantifying the effects of infected co-culture supernatants on ZO-1 localization and PD-L1 expression in the oBRB model. Data are presented as mean ± SEM from a representative experiment (n=12), repeated three times with similar results. Statistical analysis by one-way ANOVA: ns P>0.05, *P<0.05, **P<0.005 as indicated on the graphs. **(E)** Confocal Z-stack and orthogonal views of the oBRB model reveal reduced PD-L1 expression and ZO-1 dislocation in response to treatment with infected co-culture supernatants. The dashed line indicates the apical cell side and arrowheads diffuse ZO-1 localization. **(F)** MFI analysis of confocal images quantifying the effects of direct *T. gondii* RH infection on PD-L1 expression in the oBRB model. Data are presented as mean ± SEM from a representative experiment (n=9), repeated twice with similar results. Statistical analysis by one-way ANOVA: ***P<0.0005 as indicated on the graphs. **(G)** Confocal images showing the effects of direct *T. gondii* RH infection on PD-L1 and ZO-1 expression in the oBRB model. Infections were performed with the multiplicity of infection (MOI) indicated in the figure. The "lower compartment" condition represents the placement of 3x10^5 *T. gondii* RH tachyzoites in the bottom compartment of the oBRB model without direct cell contact. Representative images are shown from one of three independent replicates. **(H)** Confocal images showing the effects of supernatants from *T. gondii* RH and Me49-infected co-cultures treated with a protease inhibitor cocktail on PD-L1 and ZO-1 expression in the oBRB model. Representative images are from one of three independent replicates. **(I)** MFI analysis of confocal images illustrating the effects of protease inhibitor-treated *T. gondii* RH and Me49 supernatants on PD-L1 expression in the oBRB model. Data are presented as mean ± SEM from three independent experiments (n=12). Statistical analysis by one-way ANOVA: ns P>0.05, ***P<0.001, ****P<0.0001 compared to DMEM control, ##P<0.01, ###P<0.001 compared to inhibitor-untreated parasite supernatant.

Local parasitic proliferation in retinal cells during the acute phase of OT may trigger the release of soluble factors that influence retinal barriers, potentially modulating immune cell infiltration and activation. The exact routes by which the parasite enters the retinal space remain under debate, but direct infection of the retinal pigment epithelium (RPE) has not been observed so far *in vivo*. Therefore, our study focused on the indirect effects of retinal infection on the oBRB. To examine these early stages of infection, we developed a simplified model to explore interactions between retinal glial and microglial cells and used our established oBRB model (9, 12) (**Figure 3A**). This oBRB model consisted of 8-week differentiated RPE cells cultured on transwells. To complete this model, astrocytes, microglia, and Müller cells were co-cultured at a 1:1:1 ratio, infected with *T. gondii* RH or Me49 tachyzoites at a 1:1 MOI for 20 hours, and the resulting supernatants applied to the oBRB model for 24 hours. PD-L1 and ZO-1 expression and localization on the oBRB model were then assessed using confocal microscopy. ZO-1 is an intracellular membrane-associated chaperone protein that plays a central role in the stability and regulation of tight junctions.

RPE cells were differentiated using a specific culture media, MEM-Nicotinamide (MEM-Nic), while co-cultures were incubated in DMEM, which has a different composition and higher glucose and serum concentrations. To ensure these differences did not affect PD-L1 or ZO-1 expression, we first confirmed that the transition from MEM-Nic to DMEM had no significant impact on these markers (**Figures 3B, D**). Treatment of RPE cells with supernatants from RH-infected co-cultures significantly reduced both PD-L1 and ZO-1 labeling (**Figure 3B**) compared to non-infected controls, as also shown by MFI measurements (**Figure 3D**). Supernatants from Me49-infected co-cultures induced a similar decrease in PD-L1 expression but had no effect on ZO-1 (**Figures 3C, D**). Z-stack images and orthogonal views also revealed a clear reduction in PD-L1 apical localization (dashed line) and perinuclear staining, while ZO-1 localization became diffuse (arrowhead) under infected conditions, suggesting ZO-1 membrane delocalization rather than protein degradation (**Figure 3E**). These results demonstrate that soluble factors from *T. gondii*-infected co-cultures significantly reduce PD-L1 expression in the oBRB model, with a similar effect observed for both RH and Me49 strains, indicating a strain-independent response.

As most cytokines would be expected to upregulate PD-L1, we hypothesized that a parasite-derived factor may be responsible for the observed decrease. To test this, we investigated whether *T. gondii* infection directly impacts PD-L1 expression in RPE cells within the oBRB model. RPE cells were infected with RH tachyzoites at 1:10 and 1:1 MOIs corresponding respectively to 30–000 and 300–000 tachyzoites per well. In an additional condition, parasites were suspended in the lower chamber medium without direct contact with the cells. Confocal imaging revealed that at an MOI of 1:1, PD-L1 expression was significantly reduced compared to the 1:10 MOI condition (**Figures 3F, G**). Notably, this effect was not dependent on direct contact between parasites and cells, as it was also observed when parasites were only present in the lower chamber (**Figures 3F, G**). To further confirm that secreted or excreted parasitic factors alone could reduce PD-L1 expression, we incubated 10^7^ *T.gondii* RH or Me49 tachyzoites in DMEM for 24 hours and applied the resulting supernatants to the oBRB model for 20 hours. Confocal microscopy analysis revealed that both *T. gondii* RH and Me49 tachyzoites supernatants decreased PD-L1 expression to a similar extent as infected co-culture supernatants (**Figures 3H, I**). We showed above that infection does not impact PD-L1 mRNA expression, suggesting that PD-L1 degradation or redistribution occur rather than changes at the transcriptional level.

Given these findings and previous reports that *T.gondii* secreted/excreted proteases are capable of disrupting tight junctions, facilitating paracellular passage (14), we hypothesized that parasitic proteases might also mediate PD-L1 degradation. To test this, we treated parasite supernatants with a protease inhibitor cocktail prior to incubation of the oBRB model. This treatment neutralized the effect of the supernatants, restoring PD-L1 expression levels, independently of the parasite strain (**Figures 3H, I**).

These results confirm that parasite-derived proteases contribute to PD-L1 degradation by cleaving membrane-bound PD-L1 or promoting its internalization and degradation, thereby reducing PD-L1 expression on RPE cells in the oBRB model.

### Temporal patterns of PD-L1 expression and Müller cell activation in mouse retinas

*In vitro* experiments showed that *T. gondii* infection and interferon stimulation significantly modulate PD-L1 expression in retinal cells, with a prominent effect on Müller cells. To translate these findings to an *in vivo* context, we investigated the role of interferons in modulating PD-L1 expression and immune cell behavior within the retina in our established mouse model of OT. To investigate how type I and III interferon responses shape retinal immune dynamics *in vivo*, we intravitreally treated mice with neutralizing antibodies against IFN-β and IFN-λ2/3, along with *T. gondii* Me49 tachyzoites. We assessed the effect of infection and IFN-β or IFN-λ2/3 neutralization on PD-L1, GFAP (glial and activation marker) and CD11b (macrophage and microglial marker) expression in the mouse retina, using confocal microscopy.

**Figure 4:**
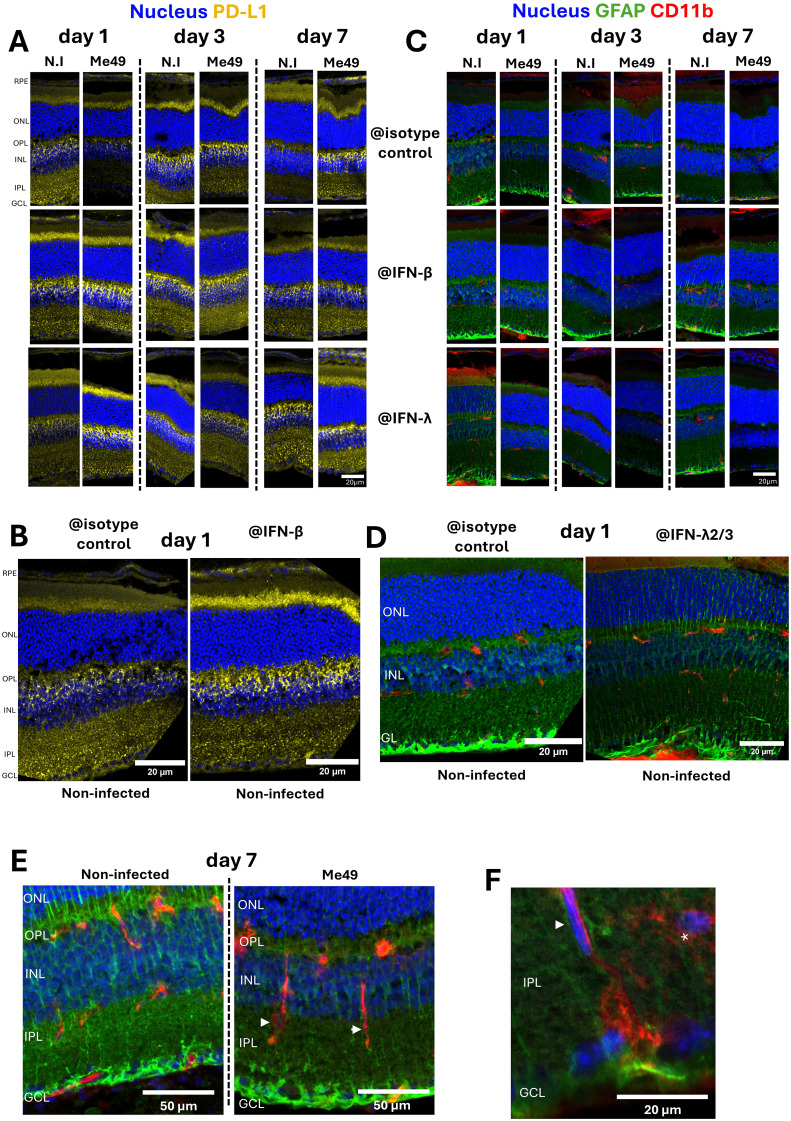
Impact of interferon neutralization on PD-L1 expression, GFAP levels, and CD11b+ cell distribution in a mouse model of ocular toxoplasmosis. **(A)** Confocal microscopy images of non-infected or *T. gondii* Me49 infected mouse retina cryosections at 1, 3, and 7 days post-injection of 20 ng of either isotype control, IFN-β, or IFN-λ2/3 neutralizing antibodies (arranged left to right for time points, top to bottom for antibody types). Retina layers are oriented from top to bottom as follows: RPE, outer nuclear layer (ONL), outer plexus layer (OPL), inner nuclear layer (INL), inner plexus layer (IPL), and ganglion cell layer (GCL). GFAP is labeled as described in green and CD11b in red. Nuclei are stained blue with Hoechst33342. **(B)** Confocal images of non-infected mouse retina cryosections at day 1 post-injection, comparing isotype control (left) and IFN-β (right) neutralizing antibody treatments. Retina layers are oriented and labeled as described. **(C)** Confocal microscopy images of non-infected or *T. gondii* Me49 infected mouse retina cryosections at 1, 3, and 7 days post-injection of 20 ng of either isotype control, IFN-β, or IFN-λ2/3 neutralizing antibodies (arranged left to right for time points, top to bottom for antibody types). Retina layers are oriented as previously described. GFAP is labeled as described in green and CD11b in red. Nuclei are stained blue with Hoechst33342. **(D)** Confocal images of non-infected mouse retina cryosections at day 1 post-injection, comparing isotype control (left) and IFN-λ2/3 (right) neutralizing antibody treatments. Retina layers are oriented and labeled as described. **(E)** Confocal Z-stack images of mouse retina cryosections at day 7 post-injection, comparing non-infected and *T. gondii* Me49-infected retinas treated with isotype control antibodies. Retina layers are oriented and labeled as described. **(F)** Confocal Z-stack image of *T. gondii* Me49-infected, isotype control antibody-treated retina sections at day 7 post-injection. Retina layers are oriented and labeled as described.

Previous work (15) suggested that retinal inflammation peaks at day 7 post-infection. Consistent with these findings, while no major infection effects were evident at days 1 and 3 (when comparing non-infected and infected condition at each day), PD-L1 expression was markedly upregulated by day 7 post-infection (**Figure 4A**). Furthermore, IFN-β neutralization in non-infected retinas enhanced PD-L1 labeling on day 1, as highlighted on the magnified view (**Figure 4B**), and to a lesser extent on day 3. By contrast, IFN-λ2/3 neutralization increased PD-L1 expression in infected retinas on days 1 and 7, compared to both infected isotype controls and non-infected IFN-λ2/3-neutralized eyes, as observed in the complete set of images (**Figure 4A**).

In agreement with our *in vitro* findings, PD-L1 expression was more prominent in Müller cells than in RPE cells or astrocytes. Müller cells, as the most abundant macroglial cells in the retina, play a central role in photoreceptor homeostasis and survival. Under homeostasis, GFAP expression was localized at Müller cell endfeet in the ganglion cell layer (GCL), with few fibers extending through the inner nuclear layer (INL) and outer nuclear layer (ONL). Activation of Müller cells, typically triggered by stress stimuli, is identifiable by the expression of GFAP in the whole cell length forming fibers extending through all retinal layers. Confocal microscopy showed that infection induced Müller cell activation on days 1 and 3 post-infection, but activation levels appeared to normalize by day 7 (**Figure 4C**). Interestingly, in the absence of infection, IFN-β and IFN-λ2/3 neutralization already led to Müller cell activation compared to isotype controls (**Figure 4C**), with particularly notable activation on days 1 and 7, as highlighted in this magnified view (**Figure 4D**). By contrast, in infected retinas, neutralization of IFN-β and IFN-λ2/3 had the opposite effect, reducing infection-induced Müller cell activation. (**Figure 4C**). With IFN-λ2/3 neutralization, this effect became apparent by day 1, peaked at day 3, and declined by day 7. These findings show that IFN-β and IFN-λ2/3 are necessary to maintain Müller cell homeostasis in non-infected retinas. By contrast, Müller cell activation in response to infection is enhanced by IFN-β and IFN-λ2/3, either directly or indirectly.

Müller cell activation in response to infection was further associated with microglial activation and migration, which displayed elongated CD11b-positive cells within the IPL of infected retinas (arrowheads), as showed by confocal z-stack imaging on day 7 (**Figure 4E**). Microglia located within the IPL normally exhibit highly ramified structures (asterisk) whereas activation under inflammatory conditions induces morphological changes and migration (arrowhead) (**Figure 4F**). While CD11b does not distinguish between microglia and macrophages, cell morphology strongly suggested that these cells were microglia (**Figures 4E, F**).

In summary, we demonstrated that PD-L1 expression peaked at day 7 post-infection at the height of inflammation. According to the localization of PD-L1 labeling, Müller and RPE cells appear mostly responsible for this upregulation. Furthermore, IFN-β and IFN-λ2/3 play different roles, acting respectively on non-infected and infected retinas across early and later time points. Finally, infection triggers microglial activation and migration together with Müller cell activation, further highlighting their importance during OT.

## Discussion

In this study, we aimed to elucidate the dynamics of PD-L1 (CD274) expression during OT and investigate the roles of type I and III interferons in regulating PD-L1 expression in the retina. PD-L1 is critical in maintaining immune privilege, particularly in the retina, where T cell-mediated immunity is essential for controlling *T. gondii* cysts and driving anti-parasitic responses (16). Interferons are potent inducers of PD-L1 expression, primarily due to the IRF1 binding site located on the promoter of the *CD274* gene (17). Consistent with that, our findings demonstrate that type I and II interferons, but not type III, effectively upregulate PD-L1 mRNA expression in retinal cell types such as microglia, astrocytes, Müller cells, and RPE cells. This aligns with previous studies showing that IFN-λ does not induce IRF1-dependent chemokines, such as CXCL9 and CXCL10, in similar cell types (9). However, fluorescence microscopy revealed that IFN-λ1 does upregulate PD-L1 localization at the cell membrane on Müller cells, suggesting the presence of alternative, cell-type-specific pathways that enhance membrane expression without a corresponding increase in transcription. Previous studies showed that PD-L1 is regulated through post-translational modifications, such as poly-glycosylation, which stabilizes PD-L1 at the membrane, protecting it from endocytosis and subsequent proteasomal degradation (18).

Notably, Müller cells, the most abundant glial cells in the retina, showed the highest baseline PD-L1 expression at both the mRNA and protein levels, responding robustly to interferon stimulation. These findings point to a more prominent immunoregulatory role for Müller cells in the retina than previously recognized. Together with microglia and astrocytes, Müller cells are a major part of the non-neuronal component of the retina and are primarily responsible for maintaining retinal homeostasis and supporting neuronal function. However, this homeostasis is disturbed when *T. gondii* replicates within the retina, infecting diverse cell types, including both resident and infiltrating immune cells (19, 20). Under inflammatory conditions, Müller cells actively participate in immune responses, exhibiting antigen-presenting cell (APC) behavior and potentially modulating to local immune environment (21). Microglia, the central nervous system’s primary resident immune cells, balance pro- and anti-inflammatory functions during retinal inflammation (22, 23), while astrocytes release various inflammatory mediators (10, 24). Additionally, astrocytes and Müller cells contribute to the disruption of the inner blood-retinal barrier (iBRB) during various inflammatory conditions (10).

To assess the impact of pro-inflammatory factors released by *T. gondii*-infected microglia, astrocytes, and Müller cells on the outer blood-retinal barrier (oBRB), we hypothesized that cytokines produced during infection could modulate oBRB function and induce PD-L1 expression to dampen immune activation. As expected, exposure of the oBRB to supernatant from infected retinal cell co-cultures destabilized ZO-1, a key tight junction protein, consistent with previous reports (9, 14). However, contrary to our expectations, supernatant from infected co-cultures significantly reduced PD-L1 expression on the oBRB. Since most inflammatory and anti-inflammatory cytokines increase PD-L1 expression (18), this finding suggests that *T. gondii* may release soluble factors that specifically suppress PD-L1. Indeed, our data indicate that both active infection and parasite-conditioned media decrease PD-L1 expression in the oBRB model. Recent studies have shown that the *T. gondii* genome encodes 49 distinct metallopeptidases (25) which can disrupt tight junctions in Madin-Darby canine kidney (MDCK) cells (14). Therefore, we investigated the effect of protease inhibitors on PD-L1 expression and found that protease inhibition partially restores PD-L1 expression at the oBRB. This suggests a novel protease-dependent mechanism through which *T. gondii* may downregulate PD-L1 to modulate immune responses. The exact nature and origin of this protease remain unclear; it may be secreted directly by the parasite or produced by RPE cells in response to a parasitic factor. The hypothesis of RPE-derived protease secretion is supported by the observation that infection-induced decrease in PD-L1 expression appears to be specific to RPE cells. However, this specificity might also reflect the lower baseline PD-L1 expression in RPE cells, making them more susceptible to downregulation compared to other cell types. It is also worth considering that this effect could result from alternative parasite-driven mechanisms, such as STAT1 inhibition via the *T. gondii* TgIST effector (26, 27), or extracellular vesicles carrying long non-coding RNAs (lnc-RNAs) or immune-modulating effectors, as observed in *Plasmodium* and other apicomplexan parasites (28). Since both type I RH and type II Me49 strains similarly reduced PD-L1 membrane localization, the involvement of strain-specific virulence factors such as GRA15 or GRA16 is unlikely (29, 30).

Our *in vitro* results suggest a potential immune evasion mechanism in which *T. gondii* downregulates retinal PD-L1 expression. This reduction may exacerbate local inflammation and contribute to BRB disruption, especially in the early stages of infection. By contrast, our *in vivo* mouse model showed upregulation of PD-L1 expression, consistent with the study by Charles et al. (3). These contrasting observations highlight the complexity of PD-L1 regulation. A key limitation of our *in vitro* model is the lack of interferons and other cytokines secreted by infiltrating immune cells. This is particularly relevant given that Charles et al., using IFN-γ knockout mice, demonstrated that IFN-γ plays a central role in driving PD-L1 expression, yet they also showed that certain cell populations retained elevated PD-L1 expression in the absence of IFN-γ, suggesting the involvement of additional regulatory pathways. In our *in vitro* experiments, we were able to dissect the complex interplay between interferons and retinal cells and found that both IFN-β and IFN-λ significantly contribute to PD-L1 regulation, particularly in Müller cells, which exhibited the highest levels of PD-L1 expression. These findings underscore the critical role of all three interferon types in regulating retinal PD-L1 expression.

It is important to consider that due to the low parasite load in the mouse retina, secreted proteases likely act more locally or at earlier stages than those assessed in our study. Additionally, differences in PD-L1 degradation between human and murine systems may be significant and cannot be ruled out. These hypotheses warrant further investigation to better understand the temporal and spatial dynamics of protease activity during infection. Finally, while our study did not specifically focus on MHC expression in Müller cells, recent research underscores their potential role as antigen-presenting cells (APCs), expressing both MHC class I and II molecules alongside co-stimulatory factors (21). This observation is particularly significant, as MHC expression is crucial for the effective interaction of PD-L1 with T cells. A recent study on autoimmune uveitis further reinforced the central role of Müller cells, demonstrating their dual function in recruiting immune cells via chemokine secretion and regulating immune responses through the expression of immunomodulatory ligands and receptors (31), which corroborates our previous findings that Müller cells secrete high levels of chemokines in response to *T. gondii* infection and interferon stimulation (9) and confirm their central role in retinal immune modulation.

## Conclusion

Our study provides new insights into the regulation of PD-L1 in retinal cells during OT, highlighting the distinct roles of type I and III interferons in this context. Müller cells emerge as key regulators of retinal immune homeostasis, particularly in their response to interferon signaling. The parasite T. gondii downregulates PD-L1 in the oBRB by a protease-dependent mechanism, potentially contributing to immune evasion and inflammation in retinal infection. Further investigations are warranted to fully elucidate retinal immune networks and PD-L1 regulation mechanisms, offering potential targets for therapeutic interventions in OT and other retinal inflammatory diseases.

## Data Availability

The original contributions presented in the study are included in the article/**Supplementary Material**. Further inquiries can be directed to the corresponding author.

## Supplementary material

Supplementary Data Sheet 1 Raw data, uncropped western blot images.

Supplementary Data Sheet 2 Raw data, excel table with MFI measurements, qPCR folds, WB folds used to make the graphs of this study.
