# Supplementary material for: Interferons and Toxoplasma gondii shape PD-L1 regulation in retinal barrier cells: the critical role of proteases
Source: Front Immunol. 2025 Jun 17;16:1607247. doi: 10.3389/fimmu.2025.1607247 (PMC12209286; doi:10.3389/fimmu.2025.1607247)

Blots images uncropped Figure 1

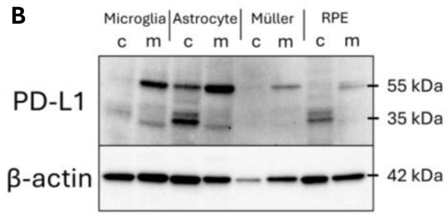

PD-L1

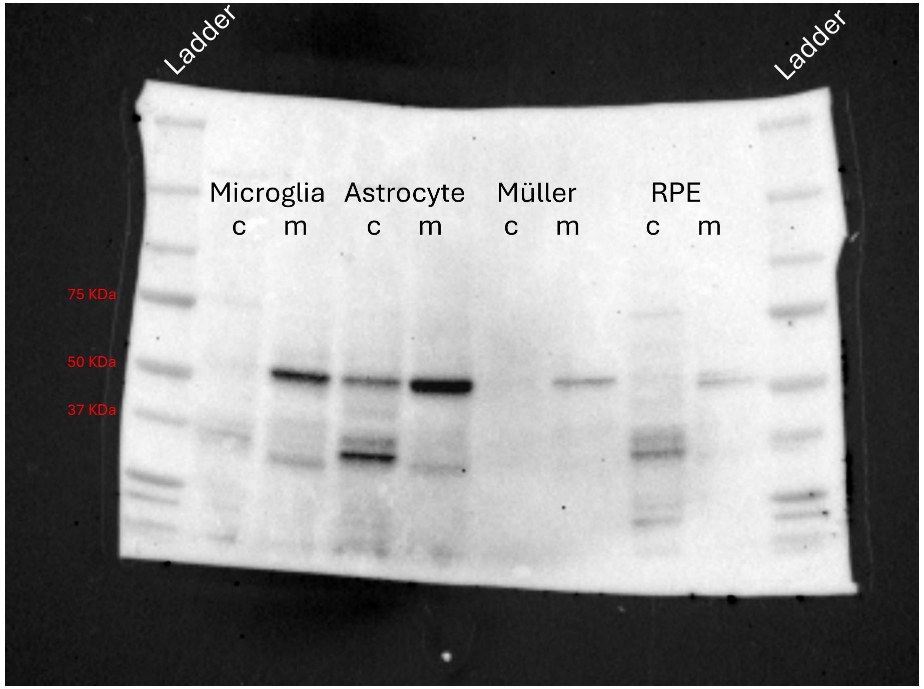

Beta-actin

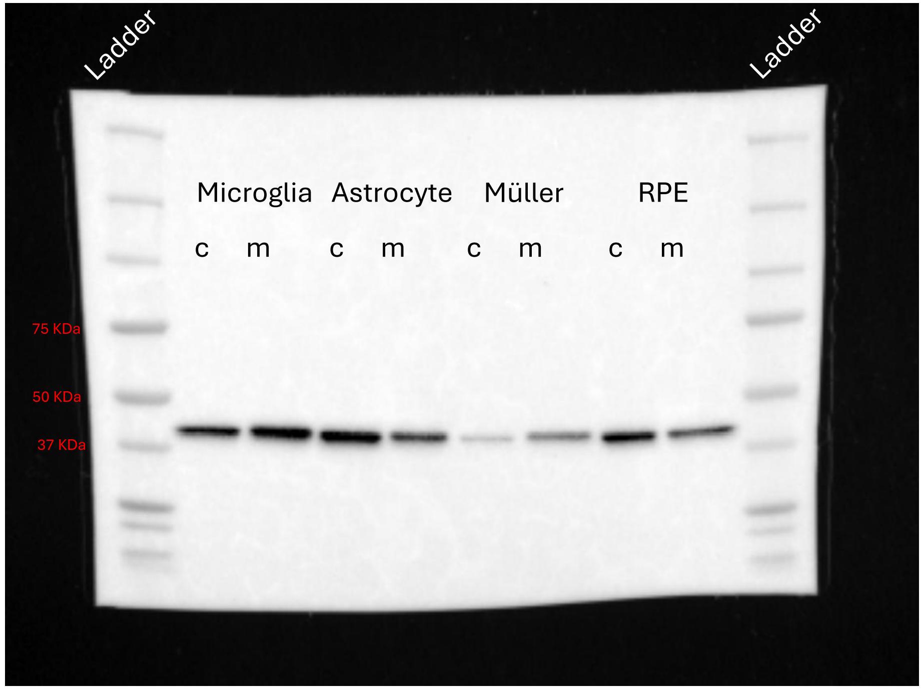

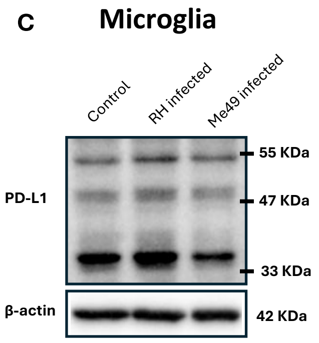
c
Microglia
Control
RH infected
Me49 infected
PD-L1
β-actin
55 KDa
47 KDa
33 KDa
42 KDa

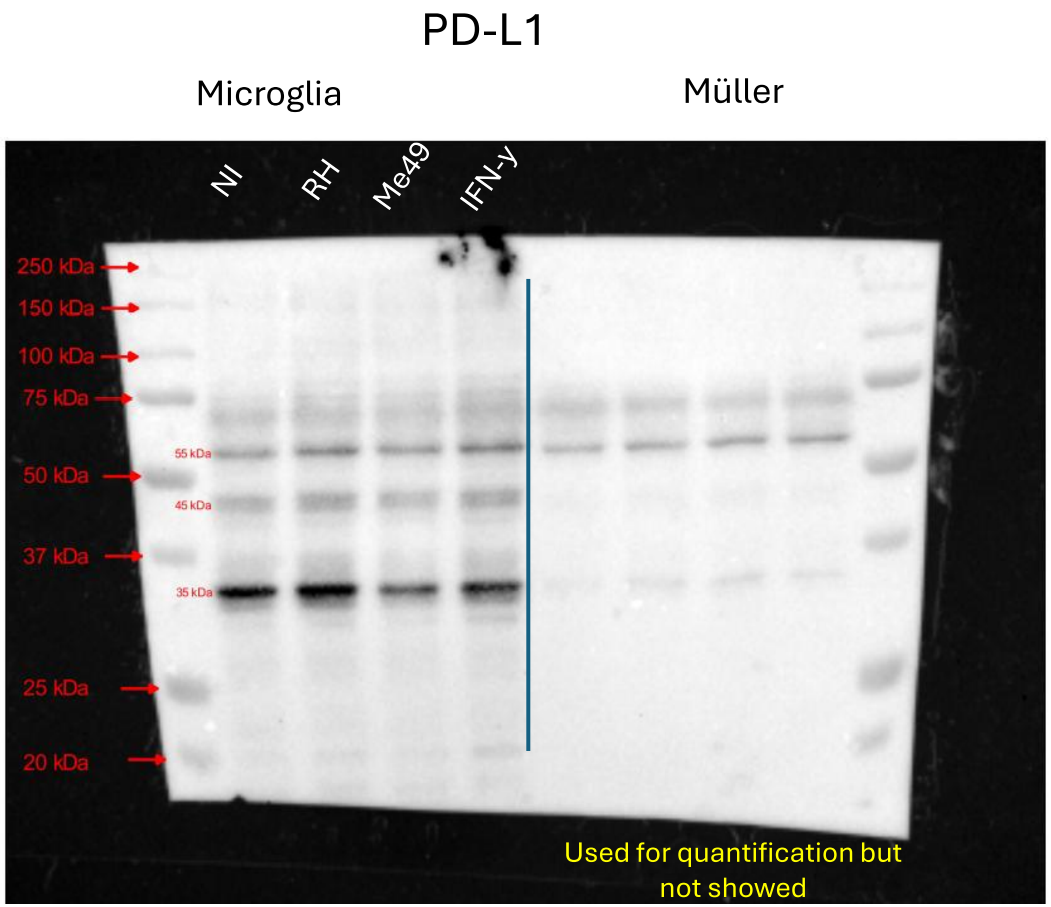
PD-L1
Microglia
Müller
NI
RH
Me49
IFN-y
250 kDa
150 kDa
100 kDa
75 kDa
50 kDa
37 kDa
25 kDa
20 kDa
55 kDa
45 kDa
35 kDa
Used for quantification but not showed

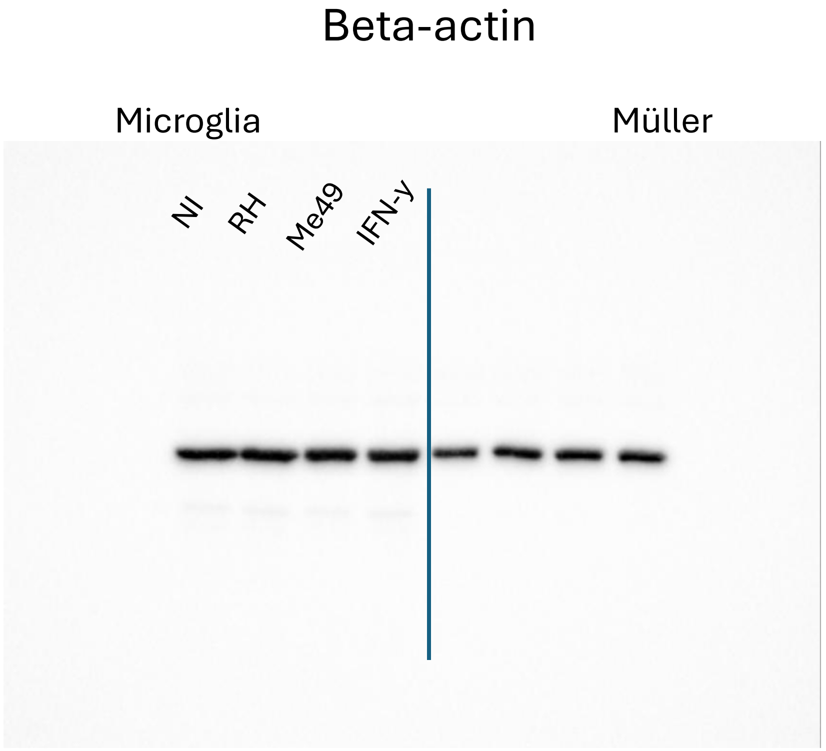
Beta-actin
Microglia
Müller
NI
RH
Me49
IFN-y

C

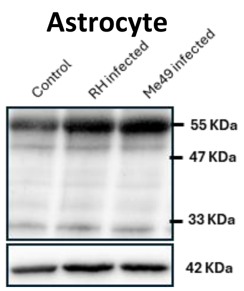
Astrocyte
Control
RH infected
Me49 infected
55 KDa
47 KDa
33 KDa
42 KDa

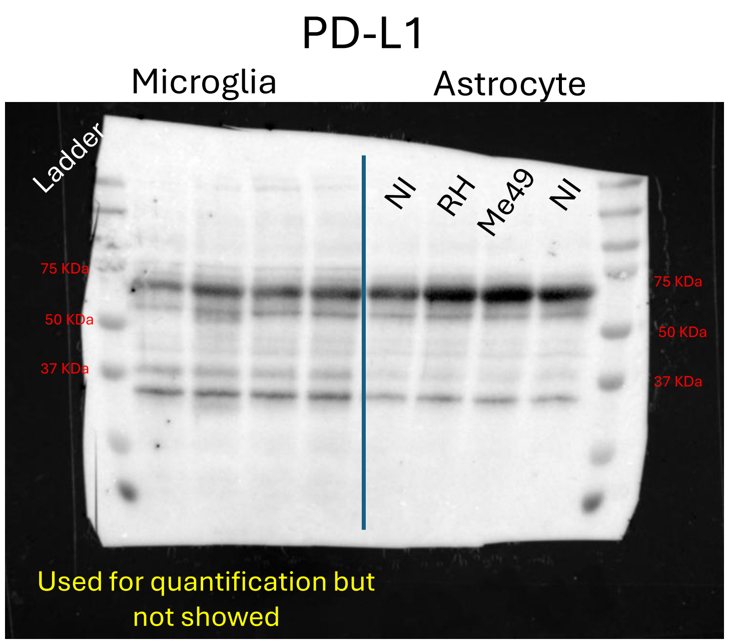
PD-L1
Microglia
Astrocyte
Ladder
NI
RH
Me49
NI
75 KDa
50 KDa
37 KDa
75 KDa
50 KDa
37 KDa
Used for quantification but
not showed

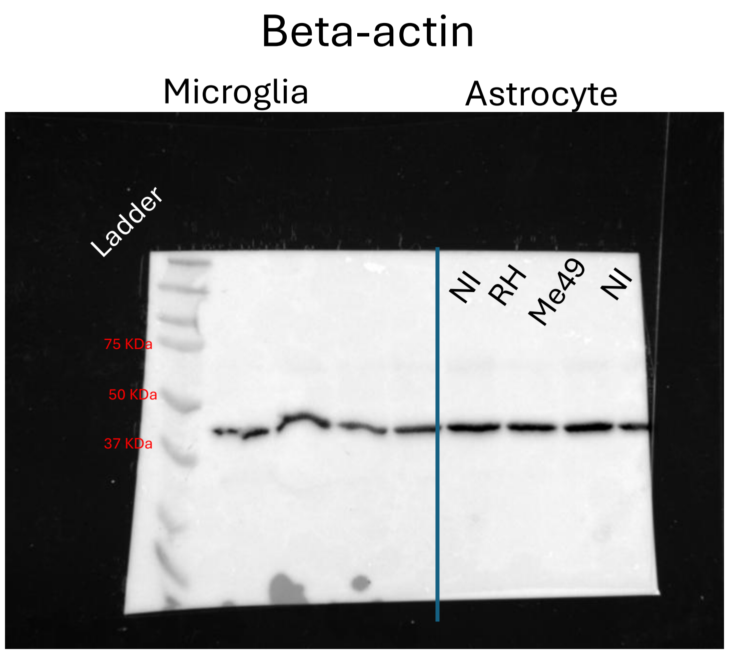
Beta-actin
Microglia
Astrocyte
Ladder
NI
RH
Me49
NI
75 KDa
50 KDa
37 KDa

c

Müller

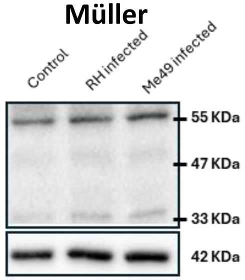

PD-L1 Müller

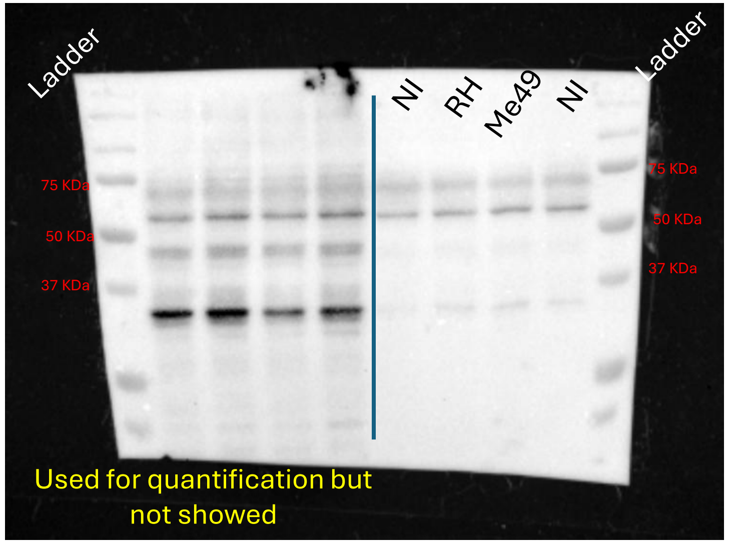

Beta-actin Müller

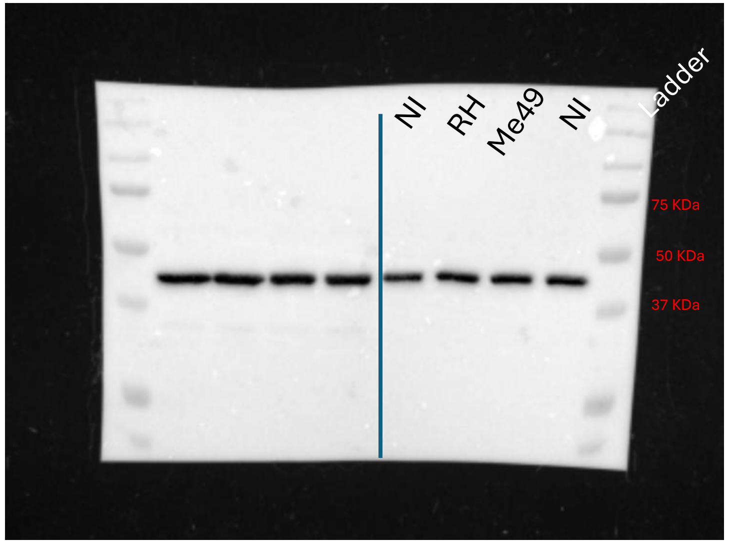

C

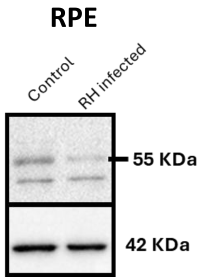
RPE
Control
RH infected
55 KDa
42 KDa

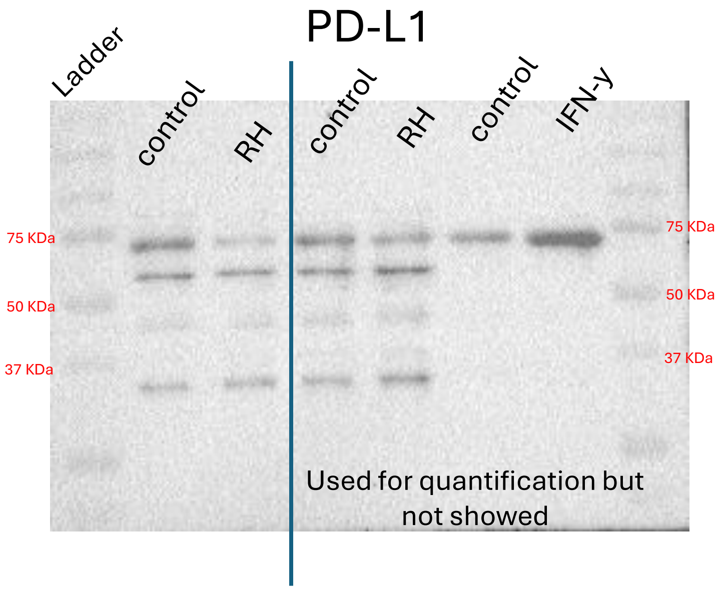
PD-L1
Ladder
control
RH
control
RH
control
IFN-y
75 KDa
50 KDa
37 KDa
75 KDa
50 KDa
37 KDa
Used for quantification but not showed

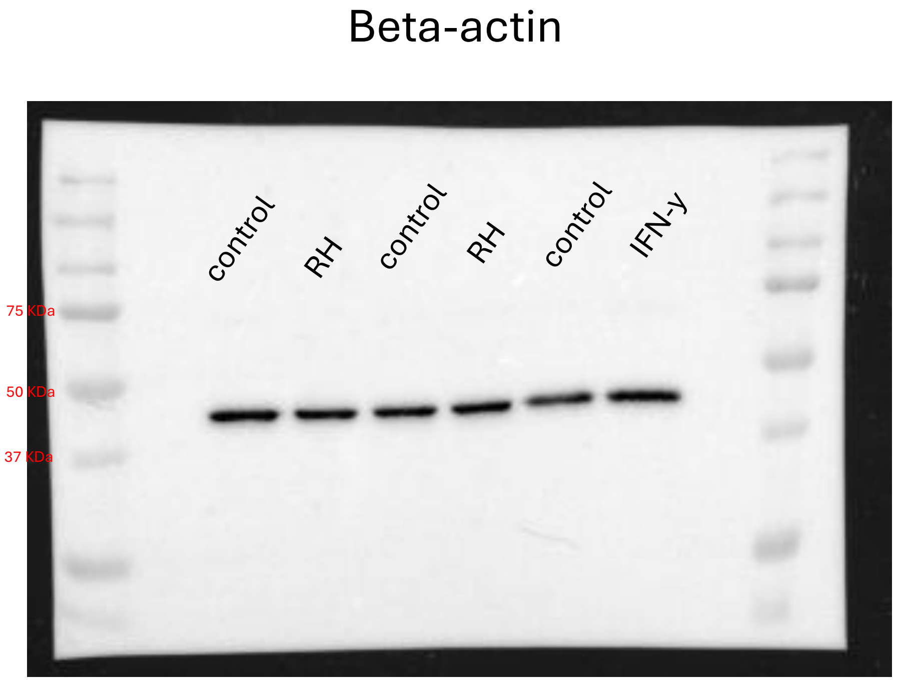
Beta-actin
control
RH
control
RH
control
IFN-y
75 KDa
50 KDa
37 KDa

E

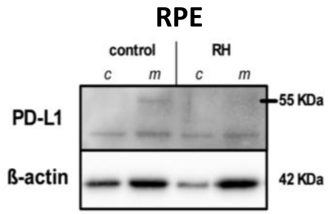

PD-L1

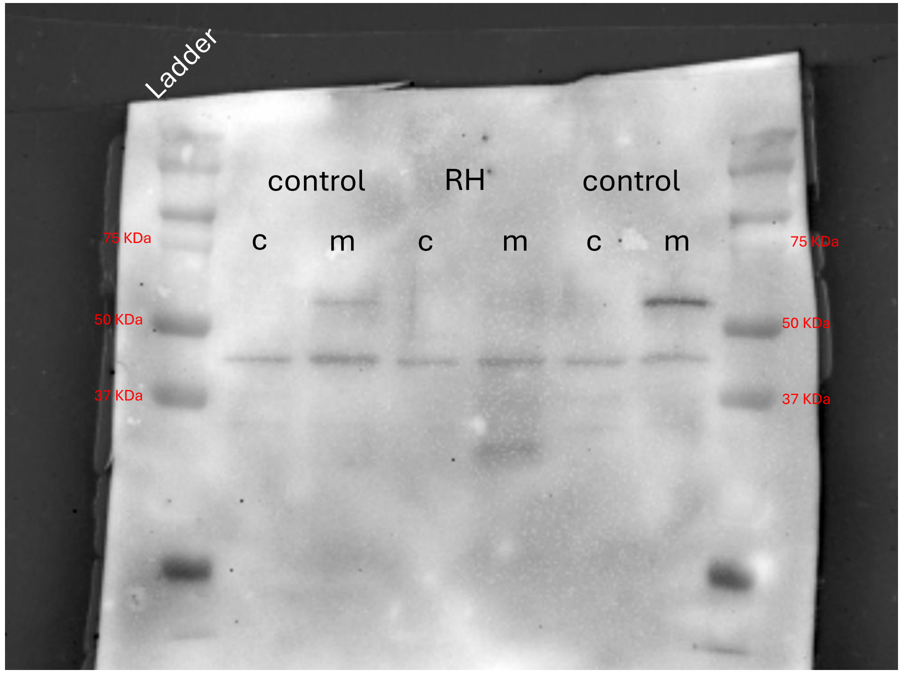

Beta-actin

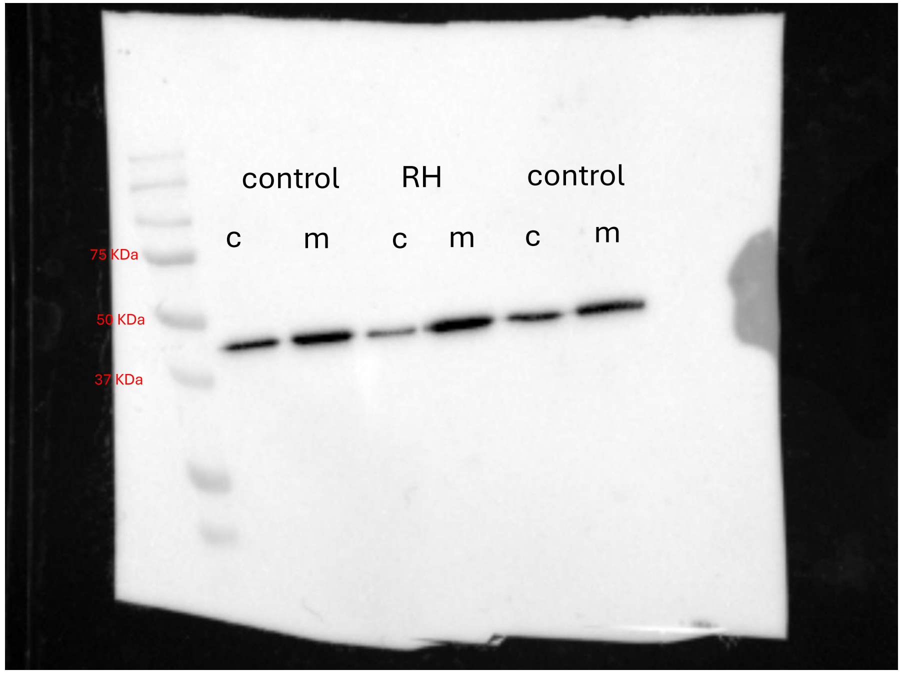

Supplement: Supplementary Data Sheet 1 — Raw data, uncropped western blot images. [file DataSheet1.pdf]
